# Meta-analysis of gene expression changes in response to radiation exposure

**DOI:** 10.1186/1471-2105-12-S7-A20

**Published:** 2011-08-05

**Authors:** John Kirtley, Eric C Rouchka, Robert M Flight, Palaniappan Sethu, John W Eaton, Robert S Keynton, The University of Louisville Research Group for Diagnosing and Mitigating Human Exposure to Radiation Using Micro-Nanotechnology

**Affiliations:** 1 Department of Computer Engineering and Computer Science, University of Louisville, Louisville, KY, 40292, USA; 2 Department of Anatomical Sciences and Neurobiology, University of Louisville, Louisville, KY, 40292, USA; 3 Department of Bioengineering, University of Louisville, Louisville, KY, 40292, USA; 4 Department of Medicine, University of Louisville, Louisville, KY, 40292, USA

## Background

Given NASA’s recent focus on long-duration space travel, potential adverse effects on astronauts of ionizing radiation need to be minimized. Levels of exposure for astronauts on such flights can be high enough to cause damage to DNA, possibly causing mutation and cancer. By analyzing the mRNA expression levels of genes exposed to different doses of gamma radiation in laboratory experiments, it can be determined which genes act as biomarkers of dosage-specific radiation exposure which can then be used on lab-on-a-chip (LOC) diagnostic platforms for early detection of ionizing radiation exposure.

## Methods

Currently, our group is incorporating three genes known to be involved in double-stranded DNA damage and repair, including: p21, p53, and γH2AX. While these genes should show up in response to ionizing radiation exposure, they are not ideal biomarkers due to their lack of specificity. Therefore, by using meta-analysis, our goal is to gain further insight into additional biomarkers for radiation exposure. More than 40 publicly-available data sets appropriate to our study were obtained from the online repository, Gene Expression Omnibus (GEO) [1]. These gene expression experiments were subsequently categorized by radiation type and dose. Meta-analysis was performed using a combination of statistical tests using the Differential Expression via Distance Synthesis (DEDS) package [2].

## Results

Preliminary meta-analysis of these publicly available datasets yields potential biomarkers from the P53 signaling pathway (CDKN1A, GADD45A, MDM2, PMAIP1), stress response transcription factors (ATF3, JUN, JUNB, JUND), and cell surface receptors (CD69, CD70, CD83). Additional microarray experiments involving irradiated blood samples are underway. The analysis of both publicly available data and our own datasets will yield a broader picture of genes most sensitive to exposure of ionizing radiation for use as biomarkers on LOC diagnostic platforms for early detection of radiation exposure, leading to subsequent treatment.
